# Vulnerability and Protective Factors Associated With Suicidal Behaviour in Adolescents: A Cross-Sectional Observational Network Analysis Study

**DOI:** 10.62641/aep.2354

**Published:** 2026-10-15

**Authors:** Julián Cádiz-Rodríguez, Miguel Ángel Carrasco, Francisco Pablo Holgado-Tello, Martina Medolla

**Affiliations:** ^1^Department of Personality, Assessment and Psychological Treatment, Faculty of Psychology, National University of Distance Education (UNED), 28040 Madrid, Spain; ^2^Faculty of Psychology, Pontifical University of Salamanca (UPSA), 37002 Salamanca, Spain; ^3^Department of Methodology of the Behavioural Sciences, Faculty of Psychology, National University of Distance Education (UNED), 28040 Madrid, Spain; ^4^Faculty of Psychology and Health Sciences, Distance University of Madrid (UDIMA), 28400 Collado Villalba, Spain

**Keywords:** suicidal behaviour, adolescence, resilience, defeat, psychometric networks

## Abstract

**Background:**

Suicidal behaviour (SB) in adolescents constitutes a major public health priority. Contemporary theoretical models, such as the Integrated Motivational–Volitional Model, emphasise the role of defeat and entrapment in the emergence of suicidal ideation. However, most studies have examined vulnerability and protective factors in isolation. The present study uses psychometric network analysis to examine how defeat, entrapment, emotion regulation, bullying and cyberbullying victimisation (CBV), resilience, reasons for living (RFL), and SB are structurally related among adolescents.

**Methods:**

A cross-sectional observational study was conducted using non-probabilistic convenience sampling. The final sample comprised 1145 adolescents and young people aged 12–21 years (mean [M] = 15.46; standard deviation [SD] = 2.16), recruited from public, state-subsidised private, private, and other collaborating settings across Madrid, Salamanca, Granada, Cádiz, Seville, and Toledo. Participants completed measures of resilience, RFL, defeat, internal and external entrapment (EE), bullying and CBV, life satisfaction, cognitive emotion regulation, and SB. After prespecified data-quality screening, residual missing values were handled using multiple imputation. Descriptive, reliability, and correlational analyses were conducted in IBM SPSS Statistics, version 31, and the prespecified Extended Bayesian Information Criterion model selection (EBICglasso) network was estimated in R, version 4.3.1.

**Results:**

The network appears to be primarily organised around a motivational core comprising defeat and entrapment. The strongest regularised partial association was observed between internal entrapment (IE) and EE (weight [w] = 0.546), followed by the connections between defeat and both EE (w = 0.304) and IE (w = 0.290). SB was directly associated with defeat (w = 0.142), EE (w = 0.125), and RFL (w = -0.215). Defeat emerged as the node with the highest strength (strength = 1.508), followed by EE (strength = 1.196), resilience (strength = 0.995), and IE (strength = 0.968). Regarding bridge indices, defeat exhibited the highest bridge strength (1.508), followed by resilience (0.995), RFL (0.709), and EE (0.651).

**Conclusions:**

The findings suggest that adolescent SB is embedded in a network in which defeat and entrapment show substantial structural relevance, whereas RFL and resilience appear as protective resources. Resilience showed both high strength and bridge strength, indicating connectivity across several construct communities rather than causal or mediational effects. These results support the hypothesis-generating value of network analysis and highlight resilience, adaptive emotion regulation, and RFL as potential targets for preventive research and intervention.

## Introduction

Adolescence is a developmental period characterised by profound biological, cognitive, and socio-emotional transformation with neurobiological changes occurring in systems involved in emotion regulation, decision-making, and behavioural control [1]. This plasticity, though it facilitates learning and autonomy, gives way to a state of biopsychosocial vulnerability in which environmental demands may exceed individuals’ regulatory resources, thereby raising the risk of psychological difficulties or disorders [2, 3].

Within this context, suicidal behaviour (SB) has emerged as one of the most pressing public health concerns worldwide. Following the terminology used by the World Health Organization, suicide is one of the leading causes of death among young people, defined in that report as individuals aged 15-29 years [4]. Recent evidence also underscores the importance of improving early detection of suicide attempts, self-harm, and suicidal ideation among adolescents [5]. Given that suicidal ideation commonly emerges during adolescence, it is critical to understand the mechanisms associated with ideation and with the transition to SB [6].

An equally concerning trend is evident in Spain. According to official data from the Spanish National Institute of Statistics [7], suicide has become the leading form of death from external causes among adolescents and young adults; in 2022 alone, 341 deaths by suicide were recorded among those aged 15–29 [8]. In the context of clinical care, the 2025 ANAR Helpline Annual Report shows that this organisation assisted 6467 children and adolescents presenting with SB in that year, 1405 of whom had already initiated a suicide attempt. Moreover, SB constituted, for the fourth consecutive year, the primary concern addressed in consultations initiated directly by minors [9].

Among the contextual stressors, peer victimisation, including traditional bullying and cyberbullying, is consistently associated with suicidal ideation, self-harm, and SBs in children and adolescents [10, 11, 12]. Cyberbullying may further intensify psychological harm because of its chronicity, dissemination, and repeated exposure, with recent evidence also linking cyberbullying victimisation (CBV) to suicidal ideation in adolescents [13].

The Integrated Motivational-Volitional (IMV) Model proposed by O’Connor and Kirtley [14] provides a key theoretical framework explaining the psychological process linking victimisation to SB. According to this model, suicidal ideation emerges during a motivational phase in which experiences of defeat (the perception of social failure) give rise to feelings of entrapment (the subjective sense of being unable to escape emotional pain). Longitudinal studies have validated psychological entrapment as the most powerful proximal mediator of suicidal ideation, representing the link between interpersonal stress and the desire to die [14, 15].

However, this risk trajectory may be shaped by both vulnerability and protective factors. Emotion dysregulation can amplify risk by making it harder to manage intense affective states after social rejection [16]. In contrast, resilience and reasons for living (RFL) may protect against the transition from suicidal ideation to SB [17, 18]. Resilience is particularly relevant because it involves positive coping appraisals, problem-solving, and help-seeking resources that may buffer the impact of defeat and entrapment on suicidal risk during adolescence [17, 19, 20].

Despite the relevance of these factors, previous research has often examined them in isolation or through linear models, limiting understanding of their joint organisation and interactions. Although network analysis has been increasingly applied to the study of adolescent suicide, the issue has still only been partially addressed. Some studies have focused on emotion regulation and contextual problems [21], broad risk and protective networks in school-aged adolescents [22], and others still on more specific symptom networks, including social, emotional, and behavioural difficulties [23, 24]. These findings support the value of network approaches, but also show that centrality and association patterns depend on the variables included in the model. Therefore, studies are still needed that integrate interpersonal, motivational, and protective factors within a theoretically grounded network. Life satisfaction was examined as a complementary cognitive indicator of subjective well-being, related but not equivalent to resilience or RFL. It was included in the descriptive and correlational analyses but was not included as a node in the prespecified Red 1 network. Its potential conceptual overlap with resilience, RFL, and adaptive cognitive emotion regulation (CERQ-P) is considered in the Discussion. This is particularly important for resilience, whose structural role within adolescent suicide-risk networks has received less attention than other protective factors [17, 19, 20].

Given the multifactorial nature of SB [14, 25], linear statistical models based exclusively on isolated predictors may be insufficient to capture the complexity of the underlying factors. Psychometric network theory provides a methodological framework that estimates conditional associations among observed variables, allowing the identification of central and bridge nodes within a relational system [26, 27].

Based on this foundation, the present study aimed to analyse, through a network approach, the structure of relationships among defeat, entrapment, emotion regulation, bullying and cyberbullying, resilience, RFL, and SB in adolescents and young people. Three research questions guided the study: (1) Which unique associations among vulnerability and protective variables remain in the regularised partial-correlation network? (2) Which nodes show the highest centrality and bridge centrality within the estimated network? (3) How are resilience and RFL structurally positioned in relation to defeat, entrapment, and SB? Given the cross-sectional design, these questions were exploratory and hypothesis-generating rather than causal.

## Materials and Methods

### Study Design

A cross-sectional observational study was conducted to analyse, using psychometric network analysis, the structure of relationships among vulnerability and protective variables associated with SB among adolescents and young people. Participants were recruited through non-probabilistic convenience sampling from educational and other collaborating settings that agreed to participate. Data were collected between September 2022 and September 2025. The recorded provinces in the final analytic sample were Madrid, Salamanca, Granada, Cádiz, Seville, and Toledo.

### Participants

An initial pool of 1181 participants was considered. Eligibility required participation through a collaborating setting, age between 12 and 21 years, voluntary consent or assent, and parental or legal-guardian consent for minors. Participants who met these criteria were subsequently excluded only when records were duplicate or internally inconsistent or when more than 20% of the assessment data were missing. Thirty-six records met one or more of these data-quality exclusion criteria, resulting in a final sample of 1145 participants. The sample comprised 635 girls (55.5%), 494 boys (43.1%), 11 participants who selected the predefined response option other (1.0%), and five participants for whom gender was not recorded (0.4%). The other category was not a data-entry error; these records passed the same data-quality criteria and were retained. Participants ranged in age from 12 to 21 years (mean [M] = 15.46; standard deviation [SD] = 2.16). Educational stage, province, and type of setting are reported in Table 1.

**Table 1.** **Baseline characteristics of the final analytic sample**.

Characteristic	N (%) or summary
Final analytic sample	1145 (100.0%)
Age, years	M = 15.46; SD = 2.16; range = 12–21
Gender: girls	635 (55.5%)
Gender: boys	494 (43.1%)
Gender: other	11 (1.0%)
Gender: not recorded	5 (0.4%)
Educational stage	Compulsory secondary education: 532 (46.5%)
	Baccalaureate: 467 (40.8%)
	Vocational education and training: 70 (6.1%)
	Undergraduate degree: 66 (5.8%)
	Stage not specified: 7 (0.6%)
	Not recorded: 2 (0.2%)
	Primary education: 1 (0.1%)
Type of educational or collaborating setting	public: 584 (51.0%)
	State-subsidised private: 429 (37.5%)
	Private: 115 (10.0%)
	Other/clinical setting: 6 (0.5%)
	Not recorded: 11 (1.0%)
Province	madrid: 797 (69.6%)
	Salamanca: 140 (12.2%)
	Granada: 71 (6.2%)
	Cádiz: 71 (6.2%)
	Seville: 49 (4.3%)
	Toledo: 1 (0.1%)
	Not recorded: 16 (1.4%)
Socioeconomic background	Diverse socioeconomic backgrounds; individual socioeconomic status was not available for participant-level analysis

Note: M, mean; SD, standard deviation. Percentages use N = 1145 as the denominator and may not total exactly 100.0% because of rounding. Other was a predefined gender response option; not-recorded categories were retained explicitly.

### Instruments

Sociodemographic data. An *ad hoc* questionnaire was used to collect information on participants’ age and gender (girl/boy/other), educational stage, type of educational setting, and province. Other was a predefined response option rather than an error in questionnaire completion or data entry.

Traditional bullying victimisation (TBV) was assessed using the three-item traditional-victimisation subscale of the 12-item European Bullying and Cyberbullying Intervention Project Questionnaire Brief (EBCIP-QB) [28]. The brief instrument contains four three-item subscales assessing traditional victimisation, traditional aggression, cybervictimisation, and cyberaggression. Items are rated from 0 (never) to 4 (more than once a week), producing a TBV score ranging from 0 to 12; higher scores indicate greater TBV. In the present sample, internal consistency was adequate (α = 0.70; ω = 0.71).

CBV was assessed using the three-item cybervictimisation subscale of the EBCIP-QB [28]. Items are rated from 0 (never) to 4 (more than once a week), producing a CBV score ranging from 0 to 12; higher scores indicate greater CBV. In the present sample, internal consistency was satisfactory (α = 0.75; ω = 0.77).

Resilience was assessed using the Spanish version of the 10-item Connor–Davidson Resilience Scale (CD-RISC-10) [29, 30]. Items are rated from 0 (not true at all) to 4 (true nearly all of the time), producing a total score ranging from 0 to 40; higher scores indicate greater perceived resilience. In the present sample, internal consistency was excellent (α = 0.88; ω = 0.88).

RFL was assessed using the 32-item Reasons for Living Inventory for Adolescents (RFL-A) [31]. Items are rated from 1 (not at all important) to 6 (extremely important), producing a total score ranging from 32 to 192; higher scores indicate more or stronger RFL. In the present sample, internal consistency was high (α = 0.88; ω = 0.96).

Defeat was measured using the 16-item Defeat Scale (DS), developed by Gilbert and Allan [32] and adapted into Spanish by Ordóñez-Carrasco *et al*. [33]. Items are rated from 0 (never) to 4 (always); items 2, 4, and 9 are reverse scored. Total scores range from 0 to 64, with higher scores indicating a stronger perception of defeat. Internal consistency was excellent (α = 0.96; ω = 0.96).

Entrapment was assessed using Gilbert and Allan’s [32] Entrapment Scale, in its Spanish adaptation by Ordóñez-Carrasco *et al*. [33]. Items are scored from 0 to 4. Internal entrapment (IE; six items; score range 0–24) and external entrapment (EE; ten items; score range 0–40) were analysed separately, with higher scores indicating higher levels of entrapment. Internal consistency was high for both IE (α = 0.93; ω = 0.93) and EE (α = 0.93; ω = 0.93).

Life satisfaction was assessed using the Satisfaction with Life Scale [34], validated in Spanish adolescents by Atienza *et al*. [35]. This scale consists of five items assessing the cognitive component of subjective well-being, that is, individuals’ global evaluation of their own lives. Items are rated from 1 (strongly disagree) to 5 (strongly agree), producing a total score ranging from 5 to 25, with higher scores indicating greater life satisfaction. Internal consistency was good (α = 0.87; ω = 0.87).

Cognitive emotion regulation was assessed using the Spanish version of the Cognitive Emotion Regulation Questionnaire (CERQ) [36], specifically the 18-item brief version proposed by Garnefski and Kraaij [37]. Items are rated from 1 to 5. Two composite scores were used: less-adaptive cognitive emotion regulation (CERQ-N), comprising eight items and ranging from 8 to 40, and CERQ-P, comprising ten items and ranging from 10 to 50. Higher scores indicate greater use of the corresponding strategies. Internal consistency was adequate for CERQ-N (α = 0.80; ω = 0.80) and CERQ-P (α = 0.85; ω = 0.86).

SB was operationalised as a study-specific composite of the six dichotomous SENTIA-derived (SENTIA refers to the Adolescent Suicidal Behavior Assessment Scale) indicators available in the analytic database [38, 39]. The score was calculated as the sum of the six indicators (range 0–6), with higher scores reflecting endorsement of a greater number of suicidal thoughts or behaviours. This composite is not equivalent to the published seven-item SENTIA Suicidal Act/Planning subscale and is therefore described explicitly as a study-specific analytic score. Internal consistency in the present sample was adequate (α = 0.85; ω = 0.88).

### Procedure

Data were collected between September 2022 and September 2025 in participating educational and other collaborating settings.

First, educational centres were contacted to introduce the study and obtain institutional approval. In the centres that agreed to participate, families were informed about the aims and content of the research, the voluntary nature of students’ participation, and the confidentiality of the data. Written informed consent was then requested from parents or legal guardians for participants under 18 years of age. Students participated voluntarily and completed the questionnaires only if they agreed to take part and, in the case of minors, if prior family authorisation had been obtained.

The assessment was conducted in person during school hours at the participating educational centres, using an online link that provided access to the different instruments. The assessment lasted approximately 90 minutes and was conducted under the supervision of two trained researchers. Data protection, storage, and custody procedures were managed by National University of Distance Education (UNED) in accordance with applicable data protection regulations.

### Data Analysis

First, the quality of the dataset was reviewed. The initial dataset consisted of 1181 participants. After applying the post-eligibility data-quality criteria described above, 36 records were excluded, yielding a final analytic sample of 1145. The remaining missing values were handled using multiple imputation. Residual missingness was concentrated in the SENTIA variables: 35 missing values in the suicidal ideation score (3.1%) and 43 missing values in the total SB score (3.8%). Five imputed datasets were generated to reduce bias associated with information loss and to stabilise the estimates.

Second, frequencies and percentages were calculated for categorical variables. For continuous variables, distributional shape was evaluated using Shapiro–Wilk tests together with inspection of skewness and kurtosis. All continuous study variables showed statistically significant departures from normality (all *p* < 0.001); therefore, Table 2 reports medians (Mds) and interquartile ranges (IQRs) as the primary descriptive statistics, together with theoretical score ranges. Internal consistency was estimated using Cronbach’s alpha (α) and McDonald’s omega (ω). Pearson correlation coefficients were calculated to examine bivariate associations among the study variables. Statistical significance was set at α = 0.05, and all inferential tests were two-sided.

**Table 2.** **Distribution-aware descriptive statistics of the study variables**.

Variable	N	Md	IQR	Theoretical range	Shapiro–Wilk *p*
TBV	1145	2.0	3.0	0–12	<0.001
CBV	1145	0.0	2.0	0–12	<0.001
IE	1145	3.0	9.0	0–24	<0.001
EE	1145	3.0	9.0	0–40	<0.001
DS	1145	12.0	16.0	0–64	<0.001
RFL	1145	159.0	38.2	32–192	<0.001
RS	1145	24.0	11.0	0–40	<0.001
SB total score	1102	0.0	0.0	0–6	<0.001
CERQ-N	1145	18.0	8.0	8–40	<0.001
CERQ-P	1145	29.0	12.0	10–50	<0.001
Satisfaction with life	1145	18.0	6.0	5–25	<0.001

Note: Md, median; IQR, interquartile range; CERQ-P, adaptive cognitive emotion regulation; CERQ-N, less-adaptive cognitive emotion regulation; TBV, traditional bullying victimisation; CBV, cyberbullying victimisation; IE, internal entrapment; EE, external entrapment; DS, Defeat Scale; RFL, reasons for living; RS, Resilience Scale; SB, suicidal behaviour. Theoretical ranges correspond to the published scoring rules. All Shapiro–Wilk tests were significant at *p* < 0.001; Mds and IQRs are therefore the primary descriptive summaries.

Subsequently, a psychometric network analysis was conducted to explore the structure of unique associations among the variables included in the original Red 1 analysis, following the guidelines proposed by Isvoranu *et al*. [27]. The prespecified network comprised ten nodes: CERQ-N, CERQ-P, traditional and cyberbullying victimisation, internal and EE, defeat, RFL, SB, and resilience. Satisfaction with life was examined in the descriptive and bivariate analyses but was not included as a node in Red 1. The network was estimated using the Graphical Lasso algorithm with Extended Bayesian Information Criterion model selection (EBICglasso), resulting in a sparse Gaussian graphical model in which edges represent regularised partial correlations between two nodes after controlling for all other nodes. Centrality indices included strength, closeness, betweenness, and expected influence (EI). Bridge strength and one-step bridge EI were calculated across the construct communities used in the original R analysis: emotion regulation, victimisation, entrapment, defeat, RFL, SB, and resilience. Bridge strength represents the sum of the absolute edge weights connecting a node to nodes in other communities, whereas one-step bridge EI represents the signed sum of direct cross-community edges.

Descriptive, reliability, and correlational analyses were conducted using IBM SPSS Statistics, version 31 (IBM Corp., Armonk, NY,USA). Network estimation, centrality and bridge analyses, and bootstrap accuracy and stability procedures were performed using R, version 4.3.1 (R Foundation for Statistical Computing, Vienna, Austria), primarily employing the qgraph, bootnet, and networktools packages. Edge-weight accuracy was examined through non-parametric bootstrapped confidence intervals. The stability of centrality estimates was evaluated using case-dropping bootstrap procedures, and correlation-stability coefficients (CS-coefficients) were calculated for strength and EI.

## Results

### Descriptive Statistics and Correlations

Table 1 reports the requested frequencies and percentages for gender, educational stage, province, and type of educational setting in the final analytic sample. Sampling method and data-collection period are reported only in the section Materials and Methods.

All continuous study variables departed significantly from normality (all Shapiro–Wilk *p* < 0.001). Table 2 therefore presents Mds and IQRs rather than relying solely on Ms and SDs. Defeat had a Md of 12.0 (IQR = 16.0), EE 3.0 (IQR = 9.0), and IE 3.0 (IQR = 9.0). RFL had a Md of 159.0 (IQR = 38.2), and resilience 24.0 (IQR = 11.0). SB was markedly right-skewed and concentrated at zero (Md = 0.0; IQR = 0.0), which explains why its SD was larger than its M in the previous presentation.

Subsequently, Pearson correlations between the main variables of the study were calculated. A selected correlation summary involving SB and resilience is presented in Table 3. The bivariate associations exhibited a pattern consistent with the theoretical model. SB was positively associated with DS (r = 0.583, *p* < 0.001), EE (r = 0.570, *p* < 0.001), IE (r = 0.544, *p* < 0.001), and CERQ-N (r = 0.378, *p* < 0.001). Conversely, SB was negatively associated with RFL (r = -0.487, *p* < 0.001), life satisfaction (r = -0.436, *p* < 0.001), resilience (r = -0.341, *p* < 0.001), and CERQ-P (r = -0.085, *p* = 0.005).

**Table 3.** **Pearson correlation matrix for the study variables**.

Variable	1	2	3	4	5	6	7	8	9	10	11
1. TBV	-										
2. CBV	0.472***	-									
3. IE	0.199***	0.248***	-								
4. EE	0.215***	0.258***	0.855***	-							
5. Defeat	0.260***	0.261***	0.815***	0.813***	-						
6. RFL	−0.231***	−0.204***	−0.503***	−0.497***	−0.596***	-					
7. Resilience	−0.184***	−0.143***	−0.467***	−0.406***	−0.588***	0.476***	-				
8. SB total score	0.187***	0.195***	0.544***	0.570***	0.583***	−0.487***	−0.341***	-			
9. CERQ-N	0.142***	0.237***	0.556***	0.570***	0.556***	−0.254***	−0.305***	0.378***	-		
10. CERQ-P	−0.113***	−0.064*	−0.135***	−0.096**	−0.225***	0.323***	0.422***	−0.085**	0.143***	-	
11. Satisfaction with life	−0.201***	−0.219***	−0.624***	−0.616***	−0.701***	0.548***	0.527***	−0.436***	−0.400***	0.249***	-

Note: CERQ-P, adaptive cognitive emotion regulation; CERQ-N, less-adaptive cognitive emotion regulation; TBV, traditional bullying victimisation; CBV, cyberbullying victimisation; IE, internal entrapment; EE, external entrapment; RFL, reasons for living; SB, suicidal behaviour. Pearson correlations are presented. Correlations involving the total score for SB were calculated with N = 1102; all other correlations were calculated with N = 1145. *p < 0.05; **p < 0.01; ***p < 0.001.

Resilience exhibited a relevant bivariate pattern. It was negatively associated with DS (r = -0.588, *p* < 0.001), IE (r = -0.467, *p* < 0.001), EE (r = -0.406, *p* < 0.001), CERQ-N (r = -0.305, *p* < 0.001), and SB (r = -0.341, *p* < 0.001), and positively associated with life satisfaction (r = 0.527, *p* < 0.001), RFL (r = 0.476, *p* < 0.001), and CERQ-P (r = 0.422, *p* < 0.001). Because bivariate correlations do not control for the remaining variables, the primary interpretation focuses on the regularised partial-correlation network reported below. Life satisfaction was interpreted only at the descriptive and bivariate levels; it was not included in the prespecified Red 1 network reported in section Psychometric Network Analysis in the Total Sample.

### Psychometric Network Analysis in the Total Sample

Fig. 1 shows the psychometric network estimated for the total sample. The network represents regularised partial correlations, so each edge reflects a unique association between two nodes after controlling for all other nodes in the network. The densest area was concentrated around motivational-volitional processes, particularly DS, IE, and EE.

**Fig. 1.**
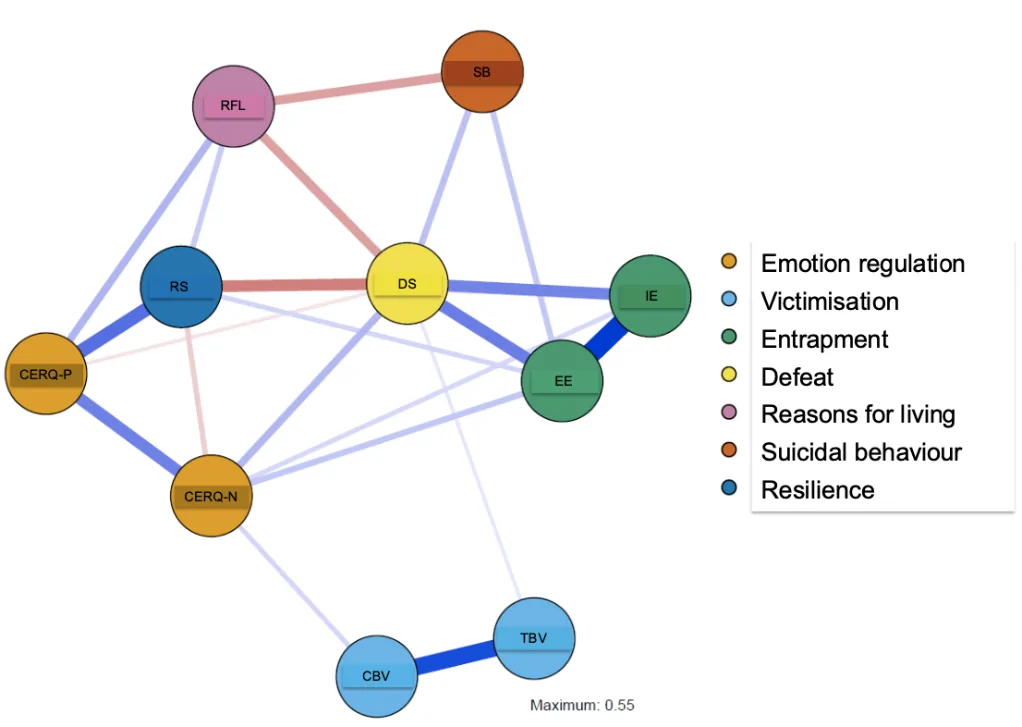
**Psychometric network estimated for the total sample**. Note: Blue lines represent positive associations, and red lines represent negative associations; line thickness reflects the magnitude of the edge weight. Network estimated using the EBICglasso algorithm. EBICglasso, Extended Bayesian Information Criterion model selection; CERQ-P, adaptive cognitive emotion regulation; CERQ-N, less-adaptive cognitive emotion regulation; TBV, traditional bullying victimisation; CBV, cyberbullying victimisation; IE, internal entrapment; EE, external entrapment; DS, Defeat Scale; RFL, reasons for living; SB, suicidal behaviour; RS, Resilience Scale.

The strongest regularised partial association was observed between IE and EE (weight [w] = 0.546), followed by TBV and CBV (w = 0.441), CERQ-P and Resilience Scale (RS; w = 0.345), DS and EE (w = 0.304), CERQ-N and CERQ-P (w = 0.294), and DS and IE (w = 0.290). These associations should not be interpreted as simple replications of the bivariate correlations; rather, they represent unique conditional associations after adjustment for the remaining variables. SB was directly connected with DS (w = 0.142), EE (w = 0.125), and RFL (w = -0.215), suggesting that SB was uniquely related to motivational risk processes and to one direct protective resource within the estimated network (Table 4).

**Table 4.** **Non-zero regularised partial correlations in the estimated network**.

Edge	Weight
IE–EE	0.546
TBV–CBV	0.441
CERQ-P–RS	0.345
EE–DS	0.304
CERQ-N–CERQ-P	0.294
IE–DS	0.290
DS–RS	−0.287
RFL–SB	−0.215
DS–RFL	−0.210
CERQ-P–RFL	0.168
CERQ-N–DS	0.163
DS–SB	0.142
CERQ-N–EE	0.128
EE–SB	0.125
RFL–RS	0.117
CERQ-N–RS	−0.108
EE–RS	0.094
CERQ-N–CBV	0.093
CERQ-N–IE	0.090
CERQ-P–DS	−0.059
TBV–DS	0.053
IE–RS	−0.043

Note: CERQ-P, adaptive cognitive emotion regulation; CERQ-N, less-adaptive cognitive emotion regulation; TBV, traditional bullying victimisation; CBV, cyberbullying victimisation; IE, internal entrapment; EE, external entrapment; DS, Defeat Scale; RFL, reasons for living; SB, suicidal behaviour; RS, Resilience Scale. Edge weights are regularised partial correlations estimated with EBICglasso. Positive values indicate positive conditional associations; negative values indicate negative conditional associations.

Resilience showed a relevant structural position within the network. It was negatively connected with DS (w = -0.287), CERQ-N (w = -0.108), and IE (w = -0.043), and positively connected with CERQ-P (w = 0.345), RFL (w = 0.117), and EE (w = 0.094). Its strongest edge was the positive association with CERQ-P, followed by the inverse association with DS. This pattern indicates that resilience was structurally connected both to protective resources and to motivational vulnerability variables. However, because the design is cross-sectional, this structural position should not be interpreted as evidence that resilience modulates, mediates, or causally alters the relation between risk and protection.

Emotion regulation variables were also connected with the motivational core. CERQ-N was linked to DS (w = 0.163) and EE (w = 0.128), whereas CERQ-P was linked to RS (w = 0.345) and RFL (w = 0.168). This pattern differentiates a vulnerability-related cluster, involving defeat, entrapment, and less-adaptive cognitive emotion regulation, from a protective cluster involving resilience, adaptive cognitive emotion regulation, and RFL.

TBV and CBV exhibited a strong direct edge with each other (w = 0.441), but weaker direct connections with the rest of the network. Thus, victimisation appeared relatively peripheral compared with motivational and protective processes in the estimated network. This does not imply that victimisation is clinically unimportant; rather, within this specific set of variables, its association with SB appears to be more indirect and structurally weaker than the associations involving DS, EE, and RFL.

Fig. 2 and Table 5 present the unstandardised centrality indices. The node with the highest strength was DS (strength = 1.508), followed by EE (strength = 1.196), RS (strength = 0.995), and IE (strength = 0.968). These results indicate that DS showed the greatest overall connectivity in the network, whereas RS was also broadly connected to the rest of the relational architecture.

**Fig. 2.**
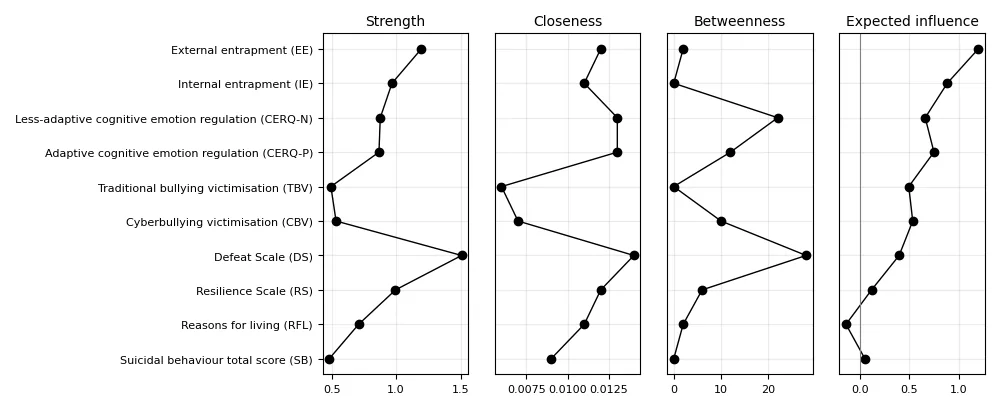
**Unstandardised centrality indices**. Note: Values calculated from the network, estimated using EBICglasso. EBICglasso, Extended Bayesian Information Criterion model selection.

**Table 5.** **Centrality indices of the estimated network**.

Node	Strength	EI	Betweenness	Closeness
CERQ-N	0.877	0.66	22	0.013
CERQ-P	0.867	0.748	12	0.013
TBV	0.494	0.494	0	0.006
CBV	0.534	0.534	10	0.007
IE	0.968	0.882	0	0.011
EE	1.196	1.196	2	0.012
DS	1.508	0.397	28	0.014
RFL	0.709	−0.139	2	0.011
SB	0.481	0.052	0	0.009
RS	0.995	0.119	6	0.012

Note: CERQ-P, adaptive cognitive emotion regulation; CERQ-N, less-adaptive cognitive emotion regulation; TBV, traditional bullying victimisation; CBV, cyberbullying victimisation; IE, internal entrapment; EE, external entrapment; DS, Defeat Scale; RFL, reasons for living; SB, suicidal behaviour; RS, Resilience Scale; EI, expected influence. The main interpretation focuses on strength and EI, as these are among the most robust indices in cross-sectional psychological networks.

In terms of EI, the highest value corresponded to EE (EI = 1.196), followed by IE (EI = 0.882), CERQ-P (EI = 0.748), and CERQ-N (EI = 0.660). RFL exhibited negative EI (EI = -0.139), which is consistent with its protective pattern of signed associations. RS showed more modest EI (EI = 0.119), probably because its positive links with protective variables and negative links with vulnerability variables offset each other in the signed index.

From an interpretative perspective, these results position DS as the most structurally connected node and EE as the node with the highest signed EI in the network. RS emerged as a node of interest because of its high strength and its edges with both protective and vulnerability-related variables. In contrast, SB occupied a less central position (strength = 0.481), suggesting that, within this cross-sectional network, it was more closely connected to specific adjacent nodes than to the overall organisation of the system.

Fig. 3 and Table 6 present bridge strength and one-step bridge EI. These indices identify nodes that connect the construct communities specified in the original R analysis: emotion regulation, victimisation, entrapment, defeat, RFL, SB, and resilience.

**Fig. 3.**
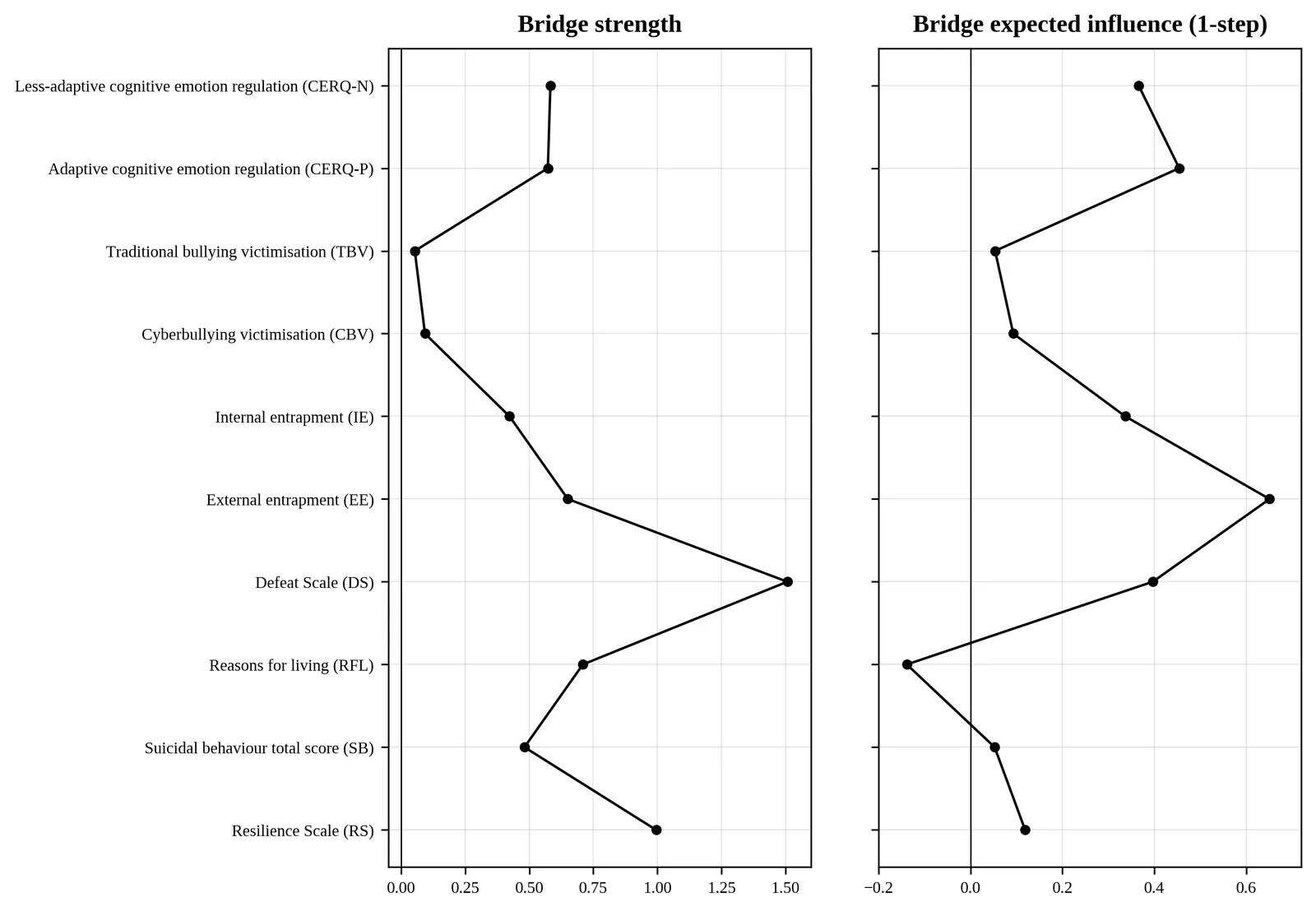
**Bridge strength and one-step bridge EI across construct communities**. Note: The two panels display bridge strength and one-step bridge EI from the original Red 1 analysis. The revised layout changes presentation only; all retained numerical values are unchanged. EI, expected influence.

**Table 6.** **Bridge indices of the estimated network**.

Node	Bridge strength	Bridge EI (1-step)
CERQ-N	0.583	0.366
CERQ-P	0.573	0.454
TBV	0.053	0.053
CBV	0.093	0.093
IE	0.423	0.337
EE	0.651	0.651
DS	1.508	0.397
RFL	0.709	−0.139
SB	0.481	0.052
RS	0.995	0.119

Note: EI, expected influence; CERQ-N, less-adaptive cognitive emotion regulation; CERQ-P, adaptive cognitive emotion regulation; TBV, traditional bullying victimisation; CBV, cyberbullying victimisation; IE, internal entrapment; EE, external entrapment; DS, Defeat Scale; RFL, reasons for living; SB, suicidal behaviour; RS, Resilience Scale. Values are reported for bridge strength and one-step bridge EI.

The highest bridge strength value corresponded to DS (1.508), followed by RS (0.995), RFL (0.709), and EE (0.651). Regarding one-step bridge EI, EE (0.651), CERQ-P (0.454), DS (0.397), and CERQ-N (0.366) were the most prominent. RFL showed negative one-step bridge EI, consistent with its signed protective connections.

These findings indicate that DS was the main structural connector across the prespecified construct communities. RS also exhibited notable bridge relevance, emerging as the second node with the highest bridge strength. This result suggests that resilience occupied a structurally connective position across several domains, without implying mediation, moderation, or causal influence.

From a clinical and interpretative perspective, the bridge position of RS is important because it was connected negatively with DS and CERQ-N and positively with CERQ-P and RFL. Resilience may therefore be considered a potential connector and a hypothesis-generating target for future longitudinal or intervention studies. However, the present cross-sectional network cannot determine whether strengthening resilience would weaken motivational risk processes or reduce SB.

Network stability analyses supported the robustness of the centrality estimates. The case-dropping bootstrap showed high stability for both strength and EI, with CS-coefficients of 0.750 for both indices. These values exceed the recommended threshold of 0.500. Non-parametric bootstrapped confidence intervals were also inspected to evaluate the accuracy of the estimated edge weights. Full bootstrap results are presented in **Supplementary Figures 1,2**.

## Discussion

This study used psychometric network analysis to examine vulnerability and protective factors associated with SB in adolescents and young people. The network was mainly organised around a motivational core of DS, IE, and EE, while RS and RFL emerged as structurally relevant protective resources. This pattern is consistent with the Integrated Motivational-Volitional Model [14] and with evidence linking defeat and entrapment to suicidal ideation in adolescents and young people, particularly highlighting entrapment as a proximal correlate of suicide risk [40, 41, 42, 43].

The strongest network edge was observed between IE and EE, followed by the edges connecting DS with both forms of entrapment. This finding suggests that perceived failure, loss of control, and psychological subordination are closely related to feeling unable to escape from aversive internal or external circumstances. Although the cross-sectional design precludes directional inferences, this structure is consistent with longitudinal and ecological momentary assessment evidence positioning defeat and entrapment as central correlates of suicide risk [40, 43].

SB was directly connected with DS, EE, and RFL in the partial-correlation network. The absence of a direct IE-SB edge after adjustment for the remaining nodes may indicate that the association between IE and SB is largely shared with defeat and EE. By contrast, EE may retain a direct edge because it captures perceived blockage in the interpersonal and contextual environment, which may be especially relevant in school-based samples. The negative SB-RFL edge supports the role of RFL as a direct protective correlate within the network [44, 45].

The findings regarding RS are particularly relevant. RS was not peripheral; instead, it showed high strength and meaningful edges with both vulnerability and protective processes. It was negatively connected with DS and CERQ-N and positively connected with CERQ-P and RFL. This position is consistent with conceptualisations of resilience as positive coping appraisals that may protect against suicide [17], as well as with reviews identifying internal and external resilience factors relevant to youth suicide prevention [20]. Prospective evidence also links positive self-perceptions, lower emotion-focused coping, physical activity, and school connectedness with lower suicidal ideation from adolescence into young adulthood [19].

The bridge results further support the structural relevance of RS. Its high bridge strength indicates connectivity across the construct communities used in the original analysis, particularly emotion regulation, defeat, entrapment, RFL, and resilience. This should be interpreted as structural connectivity, not as evidence that resilience causally links or modulates risk and protection. The finding is nevertheless clinically meaningful because protective factors against youth SB are likely to operate as interconnected systems of personal, family, school, and social resources rather than as isolated characteristics [20, 46, 47].

Regarding emotion regulation, the results illustrate two distinct relational patterns. CERQ-N was linked to DS and EE, suggesting that strategies such as rumination, catastrophising, and self-blame are associated with the motivational risk core. This is consistent with studies linking emotion dysregulation to suicidal ideation and suicide attempts among adolescents [16, 48]. Conversely, CERQ-P was connected to RS and RFL, suggesting a protective pattern associated with positive coping and the maintenance of psychological resources in the face of distress.

One finding requiring careful interpretation is the comparatively peripheral position of TBV and CBV. Although both forms of peer victimisation were strongly connected with each other, their links with the rest of the network were weaker than those of motivational and protective processes. This does not diminish their preventive relevance, given their established association with suicidal ideation, self-harm, and SB in adolescents [9, 11, 49]. Rather, they may operate as more distal contextual stressors whose relationship with SB is expressed through proximal mechanisms such as defeat, entrapment, and less-adaptive emotion regulation. Life satisfaction showed strong bivariate associations with several vulnerability and protective variables. Because it was examined descriptively and correlationally but was not included as a node in the prespecified Red 1 network, no network-edge interpretation is made for this construct. Its associations should nevertheless be interpreted cautiously because of possible conceptual and statistical overlap with resilience, RFL, and CERQ-P.

### Practical Implications

These findings have important implications for adolescent suicide prevention research and practice. The centrality of DS and EE highlights the importance of assessing subjective failure, humiliation, psychological subordination, and perceived inability to escape distress. Moreover, the structural position of RS suggests that strengthening coping resources, adaptive emotion regulation, perceived competence, and RFL could be explored as intervention targets in future longitudinal and experimental studies. Although TBV and CBV were peripheral in this network, school-based anti-victimisation programmes should be combined with interventions addressing the psychological mechanisms that may transform interpersonal adversity into suicide risk.

### Limitations and Future Research Directions

This study has several limitations. Its cross-sectional design precludes causal or directional inferences, and the results should be understood as hypothesis-generating rather than as a basis for clinical decision-making or individual risk prediction. Self-report measures may have introduced response biases, and the school-based convenience sample limits generalisation to clinical populations or adolescents at high suicidal risk. The small number of participants in the other gender category prevented meaningful subgroup analysis and should be acknowledged when interpreting gender-related generalisability. In addition, although the sample included diverse socioeconomic backgrounds, individual socioeconomic status was not disaggregated in the analysis dataset. Relevant variables such as depressive symptoms, anxiety, loneliness, family support, trauma exposure, school climate, and teacher support were not included in the network. Network stability was assessed using case-dropping bootstrap procedures, and the centrality estimates showed high stability; nevertheless, as with all cross-sectional networks, the results should be interpreted as exploratory and hypothesis-generating rather than as evidence of causal, diagnostic, or predictive effects. The study-specific six-indicator suicidal-behaviour composite is not the validated seven-item SENTIA Suicidal Act/Planning subscale and requires independent psychometric validation.

Future research should examine differences by sex, gender, educational stage, and age. Longitudinal and ecological momentary assessment designs are needed to clarify the temporal direction of associations among resilience, defeat, entrapment, and SB. School-context network studies including climate, belongingness, social support, and face-to-face or digital victimisation would also help clarify the role of bullying and cyberbullying in adolescent suicide risk.

## Conclusions

Despite these limitations, the results indicate that adolescent SB is embedded in a relational network in which defeat and entrapment constitute the motivational core with the greatest structural relevance. RS emerged as a highly connected node and as a bridge-strength node structurally connecting vulnerability and protective domains. This pattern suggests that resilience is relevant not only as a general protective factor but also as an element integrated within the network architecture of vulnerability and protection. Consequently, resilience, RFL, and adaptive emotion regulation represent promising targets for future prevention-oriented research on SB among adolescents.

## Availability of Data and Materials

The data analysed in this study are not publicly available because they contain sensitive information from minors and are subject to ethical and confidentiality restrictions. De-identified data and analysis code may be made available by the corresponding author upon reasonable request, subject to applicable ethical approval and data-protection requirements.
