# Supplementary material for: Vulnerability and Protective Factors Associated With Suicidal Behaviour in Adolescents: A Cross-Sectional Observational Network Analysis Study
Source: Actas Esp Psiquiatr. 2026 Oct 15;54(5):1365–78. doi: 10.62641/aep.2354 (PMC13714470; doi:10.62641/aep.2354)
Supplement: Supplementary file 1 [file ActEsp-54-5-1365-1378-s1.zip › ActEsp-54-5-1365-1378.docx]

**Supplementary Material**

**Supplementary Figure 1 Accuracy of regularised edge-weight estimates based on non-parametric bootstrap.**

Note: Points show the edge estimates from the original analytic sample and the corresponding bootstrap means; shaded intervals represent the uncertainty of the estimates. Edges are ordered by their original-sample values. The figure corresponds to the original Red 1 network (N = 1145).

**Supplementary Figure 2 Stability of strength and expected influence based on case-dropping bootstrap.**

Note: Lines show the average correlation between centrality estimates obtained after case dropping and the corresponding estimates in the original sample; shaded bands show the variability of these correlations. Strength and expected influence both yielded a correlation-stability coefficient of 0.750. The figure corresponds to the original Red 1 network (N = 1145).
